# Controlled sample environment for studying solid–gas interactions by *in situ* powder X-ray diffraction

**DOI:** 10.1107/S1600576720014776

**Published:** 2021-02-01

**Authors:** Paul Monceyron Røren, Kristoffer W. B. Hunvik, Vegard Josvanger, Ole Tore Buseth, Jon Otto Fossum

**Affiliations:** a Department of Physics, Norwegian University of Science and Technology, Norway

**Keywords:** sample cell, pressure, temperature, powder diffraction

## Abstract

A sample cell for powder X-ray diffraction studies with *in situ* applied pressure and control of temperature is demonstrated. The cell is based on a previously reported design and consists of a glass or quartz capillary glued into a Swagelok weld gland; this configuration can hold up to 100 bar (1 bar = 100 kPa). The cell is placed in contact with a copper plate for control of temperature between −30 and 200°C. This is achieved by Peltier elements, heat cartridges and a refrigerated circulating bath. This work mainly focuses on the temperature control system. Commissioning tests were performed in a custom-made small/wide-angle X-ray diffractometer at the Norwegian University of Science and Technology. The system is easily portable to synchrotron facilities.

## Abstract 2

This work demonstrates a sample environment suitable for studying solid–gas interactions by temperature control through thermal contact with a sample in a glass capillary, for temperature and pressure conditions of 0–100 bar (1 bar = 100 kPa) and −30 to 200°C.

## 1. Introduction   

The structural changes of materials in response to solid–gas interaction are of great importance in many areas (Michels *et al.*, 2015 ▸; Pitt *et al.*, 2011 ▸; Cheng *et al.*, 2017 ▸). In studying such changes, X-ray diffraction (XRD) is one of the most versatile tools for the investigation of the nano- and microstructures of materials, and it can accurately evaluate phase composition, defects, strain and crystallite size. Therefore, it is important to design new setups and sample cells for studies of structural changes with XRD with *in situ* control of temperature and gas pressure.

To contribute to the understanding of crystalline swelling in clay minerals in response to CO_2_, we have constructed a sample cell to study this under controlled pressure and temperature using powder XRD. Many sample cell designs exist for XRD at elevated pressures and controlled temperatures (Chupas *et al.*, 2008 ▸; Hemmen *et al.*, 2012 ▸; Jensen *et al.*, 2010 ▸; Bösenberg *et al.*, 2014 ▸; Joubert *et al.*, 2006 ▸; Scarlett *et al.*, 2008 ▸, 2011 ▸; Bon *et al.*, 2014 ▸; Koster van Groos *et al.*, 2003 ▸; Schaef *et al.*, 2012 ▸; Eu *et al.*, 2009 ▸; Becker *et al.*, 2010 ▸). Our desired temperature range is from −30 to 200°C. In this range, the clay can be dehydrated *in situ* and all the phases of CO_2_ are accessible. The X-ray chamber at the Norwegian University of Science and Technology (NTNU) has available inner dimensions W × H × D = 0.11 × 0.15 × 0.31 m, and these space limitations did not allow for temperature control by a gas stream/cryojet (Jensen *et al.*, 2010 ▸; Becker *et al.*, 2010 ▸). Radiative heating by a filament could be applied (Jensen *et al.*, 2010 ▸; Eu *et al.*, 2009 ▸); however, cooling was also desired. Our sample cell was therefore devised on the basis of temperature control by thermal contact. The elegant thin-walled glass capillary cell reported by Jensen *et al.* (2010 ▸) is easy to use, portable and versatile, it avoids the use of beryllium, and pressure can be controlled in a range from vacuum to 100 bar (1 bar = 100 kPa). Some previous designs (Becker *et al.*, 2010 ▸; Eu *et al.*, 2009 ▸) employ straight capillaries with both ends open using ferrules as seals, but this is far more cumbersome. Placing the capillary in contact with a temperature-controlled copper plate allows for both heating and cooling in the relevant temperature range, and overcomes the space limitations of NTNU’s instrument. We have employed heat cartridges to control temperature at above ambient, and Peltier elements and a circulation bath to control temperatures below ambient; in addition, strategies to avoid ice formation on the capillary were developed. This compact solution allows for easy transportation to facilities where gas control systems and circulation baths are available.

## 2. Design and experimental results   

The powdered samples are contained in capillaries mounted in a custom-made high-pressure sample cell based on the design by Jensen *et al.* (2010 ▸). Both glass (Hilgenberg GmbH: 4007605) and quartz (Hilgenberg GmbH: 4017505) capillaries with one end sealed have been used in the dedicated pressure range without capillary fracturing occurring. In the present setup the mechanical strength of the capillaries set the upper pressure limit. Typically, capillaries of 0.5 mm diameter and 0.01 mm wall thickness, cylindrical over the entire measurement range, have been used. The length of the capillary is limited by the size of the copper plate and is typically 80 mm. The capillary is glued in a Swagelok weld gland by a UV curable resin, Proformic C4001. In our experience this provides a better seal than the two-component Loctite glue described by Jensen *et al.* (2010 ▸). The Swagelok weld glands are reusable, and the glue is removed by either burning or scratching. To align the capillary as best as possible when gluing, a dedicated stand was constructed where the capillary and weld glands are aligned using two concentric holes, before applying the glue. The remainder of the design is as described by Jensen *et al.* (2010 ▸), except for the addition of Swagelok SS-QM2-B-100 and SS-QM2-D-100 quick-connects attached to the pressure line, allowing for *ex situ* manipulation of the sample and capillary without loss of atmosphere.

**Figure 1:**
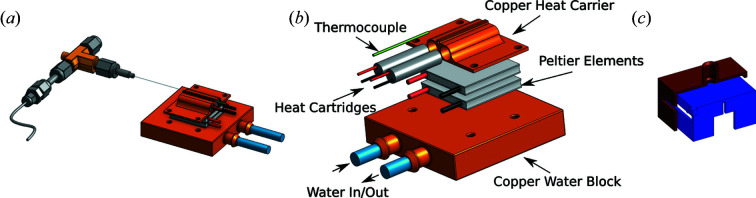
(*a*) Illustration of the sample holder mounted in contact with the temperature control assembly. The capillary is mounted perpendicular to the X-ray beam. (*b*) Expanded view of the temperature control assembly. A copper plate with dedicated holes for heat cartridges and a thermocouple is mounted on a copper water block with two Peltier elements. The assembly is clamped together by four nylon screws and thermal paste is applied between each part. (*c*) In blue is shown a 3D-printed chamber which is mainly used for blast protection. In red is the cross section of the chamber used to flow nitrogen to avoid frost formation at temperatures below freezing. Kapton or Mylar tape is used as windows where the X-rays pass through; they also force the gas to escape from the bottom.

The temperature control assembly is illustrated in Figs. 1 ▸(*a*) and 1 ▸(*b*). The copper plate ensures a good thermal transfer between the capillary and the temperature control elements. Heat cartridges are inserted with thermal paste into two dedicated holes, in the copper piece, providing a symmetric temperature profile. The copper plate is placed on a double stack of Peltier elements (APH-127-10-25-S), which are placed on the copper water block connected to a refrigerated circulating bath. Thermal paste is applied between all elements in the stack. Both the heat cartridges and the Peltier elements are powered by an ES 015-10 power supply, which is externally controlled by an overdamped PID from a *Labview* script. The setup takes 15 min to go from the system minimum (−30°C) to maximum (200°C) temperature. The consequence of using an overdamped system is a slower system but no overshoot. On average, over the entire temperature range, it takes the system about 4 s to change 1°C, and the system is faster around 30°C than at the extremities of the temperature range.

The cell is placed in contact with the copper piece by gently elevating the whole assembly up to the capillary. Thermal contact is ensured by having a well aligned capillary, which is in contact with the copper piece along the full length where powder is present. This is evaluated by first shining light onto the capillary and then estimating by the shadow whether or not the entire length of the capillary touches the copper piece. This process is repeated by imaging the capillary with X-rays passing through glassy carbon. Thermal paste could have been used to improve the thermal contact surface between the capillary and the heat transfer element, but this could interfere with the beam which in turn could be a problem in using the current low-brilliance setup at NTNU. The design of the cell has some minor shortcomings. In the present design the position can vary by 0.5 mm from one sample mounting to another. The uncertainty due to this variation is the same as the uncertainty due to the pixel size of the detector. This can be improved by making a physical guide (*e.g.* a notch in the copper plate) forcing the capillary into the same position every time. The lower scattering angles are limited by the geometry of the setup; however, the scattering limit from the center of the sample is below and outside the detector and does not limit the measurements.

To control the pressure in the capillary a *Labview*-script-controlled Teledyne ISCO 260 D syringe pump is employed, and vacuum is acquired by a rotary vane pump from Edwards. The script allows for both automated temperature and pressure control.

**Figure 2:**
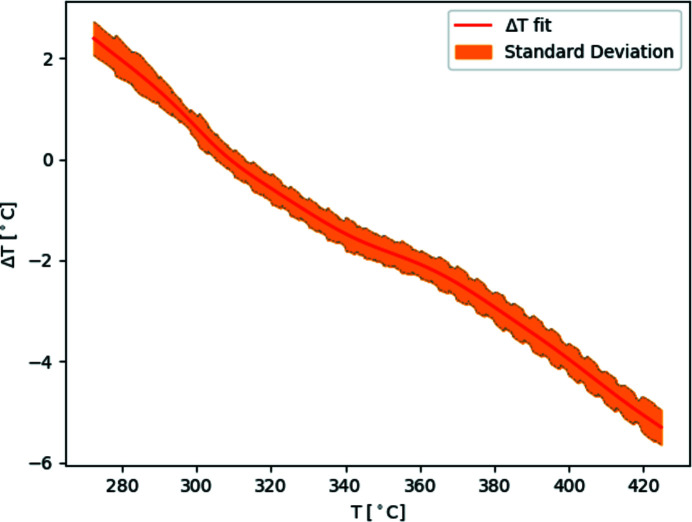
The red line is a corrected smoothing spline fit using leave-one-out cross-validation on all temperature difference measurements between the measuring point and the inside of the capillary. Several heating ramps of 0.10, 0.16, 0.25, 0.33 and 0.50°C s^−1^ as well as heating using maximum power output and a couple of stepped ramps were performed at several capillary positions and thermocouple positions. In orange, the local standard deviation of the measurements.

**Figure 3:**
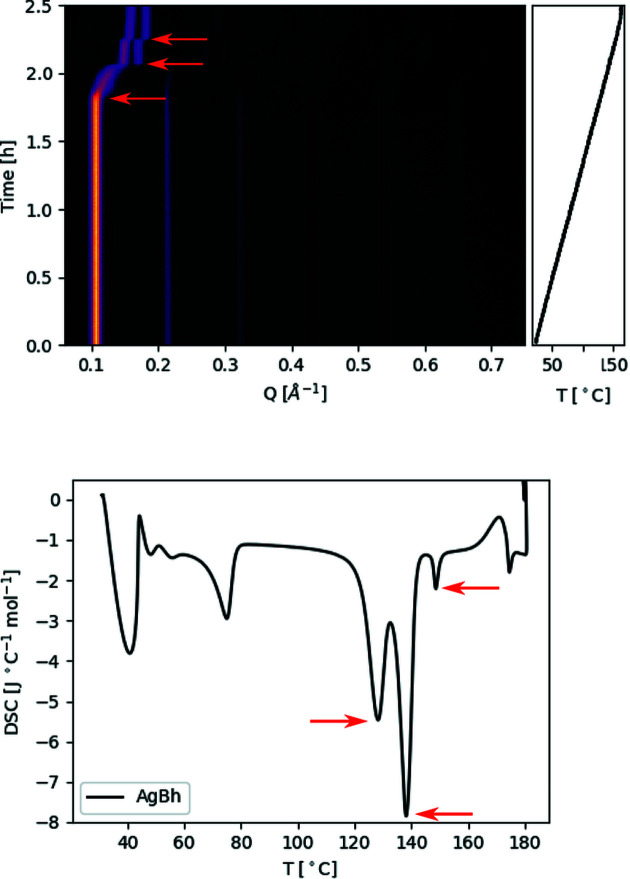
Top, a contour plot of the evolution of the Bragg reflections of AgBh as a function of time. The AgBh was subjected to a temperature ramp that went from 30 to 165°C. Bottom, the DSC of AgBh. In both figures the arrows mark the points used to calibrate the temperature curve.

To estimate the temperature in the capillary, a thermocouple mounted in a hole in the copper plate, in proximity to the contact surface, measures the temperature. The temperature is calibrated using a corrected smoothing spline fit on the temperature profile measured with a thermocouple mounted inside the capillary (Fig. 2 ▸). The temperature profiles are reproducible and independent of the temperature ramp, capillary position and thermocouple position. The correction to the temperature profile was done by estimating the measurement error by comparing the differential scanning calorimetry (DSC) of silver behenate (AgBh) and the XRD pattern change along a temperature ramp in our setup using three points, as can be seen in Fig. 3 ▸. The uncertainty in temperature is within 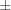
1°C. A simple linear approximation could have been used on the temperature profile, but a ‘dip’ was observed in all the curves between 300 and 370°C. The source of the ‘dip’ has not been established, but it is not a product of the PID as it was present in the maximum power curves as well. The temperature is stable within the constraints given by the hardware, the precision of the thermocouples, the power supply and the digital-to-analog converter. There is a temperature gradient across the capillary between the air and copper plate; to reduce this effect the copper heat carrier could in the future be designed to partly surround the capillary.

At temperatures near or below freezing, dew and ice formation are a challenge. To avoid dew or ice formation, several measures are taken. Firstly, a small vat containing silica gel is inserted into the X-ray chamber. Secondly, a set of two boxes is placed over the sample holder and a gentle flow of nitrogen is blown between the boxes [Fig. 1 ▸(*c*)]. This ensures a dry atmosphere around the capillary without direct interference from the gas flow. The boxes also act as protection for the detector in the eventuality of a capillary rupture.

**Figure 4:**
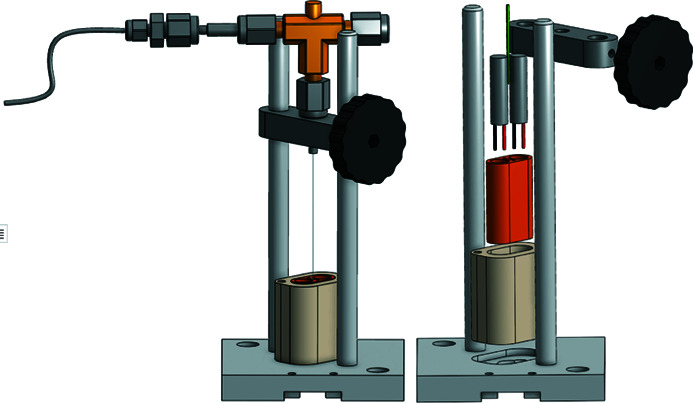
To the left is an illustration of the sample holder in an *ex situ* drying station. The sample cell with the sample in the capillary is lowered into a dedicated hole in a copper piece heated by two heat cartridges. To limit heat loss, the assembly is thermally insulated by a polyetheretherketone shroud. To the right is an expanded view of the drying station.

To increase experimental efficiency, an *ex situ* drying station, also relying on thermal contact, has been constructed (Fig. 4 ▸). This allows for heating samples at even higher temperatures, as the maximum temperature for the *in situ* setup is limited by the Peltier elements which are damaged at temperatures above 200°C. Swagelok quick-connects are used to ensure limited exposure during transfers of the samples from the drying station to the XRD setup (less than 0.1 cm^3^).

The commissioning of the sample cell was performed in a custom-made small/wide-angle X-ray scattering diffractometer at NTNU, Norway, attached to a Xenox stationary electron impact source with a copper anode, producing *K*α radiation. The scattered intensity was recorded by a 2D Dectris Pilatus3 R200K detector with sample-to-detector distance of 0.2 m, enabling us to record the full (001) Bragg diffraction ring of a powdered clay mineral. The powdered clay mineral investigated was Ni fluorohectorite (Hec).

Na-Hec with the stoichiometric composition of Na(Mg_5_Li)Si_8_O_20_F_4_ was prepared via melt synthesis according to a published procedure (Daab *et al.*, 2018 ▸), followed by annealing (6 weeks, 1045°C) to improve charge homogeneity and phase purity (Stöter *et al.*, 2013 ▸). Ni-Hec was prepared by cation exchange of Na-Hec with 0.2 *M* nickel acetate solution (>tenfold excess of the cation exchange capacity, five times). The exchanged Ni-Hec was washed five times with Millipore water.

**Figure 5:**
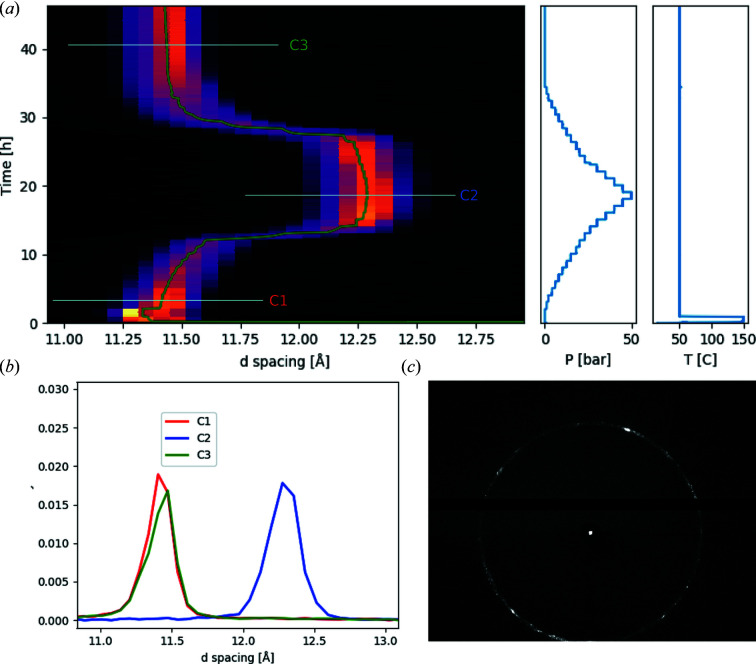
Example of a diffraction experiment on an Ni-Hec clay mineral. The sample is first dried *in situ* at 150°C under vacuum, and then brought down to a temperature of 50°C and subjected to a pressure cycle of CO_2_. (*a*) Contour plot of the evolution of a Bragg reflection as a function of time (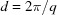), pressure and temperature given by the panels to the right. (*b*) The Bragg reflection curves C1, C2 and C3 given from three marked positions in the contour plot. (*c*) 2D diffraction pattern of the clay mineral.

In Fig. 5 ▸ experimental tests of the commissioning of the sample environment are demonstrated. Ni-Hec, in a borosilicate capillary, was first dried to remove residual water present in the sample at 150°C under vacuum, and was then subjected to a script-controlled pressure cycle of CO_2_ at 50°C, controlling the pressure between vacuum and 50 bar. For reasonable signal-to-noise ratio, each diffractogram was recorded for 5 min. Three diffractograms have been included, where the sample is exposed to 1 bar, 50 bar and 5 h of vacuum after the pressure cycle. The data demonstrate that the sample environment is able to provide the necessary conditions to study the slow dynamics of a clay mineral sample exposed to CO_2_. The sample environment is capable of drying the sample at 150°C [without overheating as indicated by Fig. 5 ▸(*a*)] under vacuum and then maintaining the sample at 50°C during the pressure cycle. The details and interpretation of the experimental data are part of a separate study (Hunvik *et al.*, 2020 ▸).

## 3. Conclusion   

We have demonstrated a sample environment suitable for studying solid–gas interactions by temperature control through thermal contact with a sample in a glass capillary, for temperature and pressure conditions of 0–100 bar and −30 to 200°C.
